# Coral-Associated Bacteria as a Promising Antibiofilm Agent against Methicillin-Resistant and -Susceptible *Staphylococcus aureus* Biofilms

**DOI:** 10.1155/2012/862374

**Published:** 2012-09-04

**Authors:** Shanmugaraj Gowrishankar, Nyagwencha Duncun Mosioma, Shunmugiah Karutha Pandian

**Affiliations:** Department of Biotechnology, Alagappa University, Karaikudi, Tamil Nadu 630003, India

## Abstract

The current study deals with the evaluation of two coral-associated bacterial (CAB) extracts to inhibit the biofilm synthesis *in vitro* as well as the virulence production like hemolysin and exopolysaccharide (EPS), and also to assess their ability to modify the adhesion properties, that is cell surface hydrophobicity (CSH) of methicillin-resistant (MRSA) and -susceptible *Staphylococcus aureus* (MSSA). Out of nine CAB screened, the ethyl acetate extract of CAB-E2 (*Bacillus firmus*) and CAB-E4 (*Vibrio parahemolyticus*) have shown excellent antibiofilm activity against *S. aureus*. CAB-E2 reduced the production of EPS (57–79%) and hemolysin (43–70%), which ultimately resulted in the significant inhibition of biofilms (80–87%) formed by both MRSA and MSSA. Similarly, CAB-E4 was also found to decrease the production of EPS (43–57%), hemolysin (43–57%) and biofilms (80–85%) of test pathogens. CLSM analysis also proved the antibiofilm efficacy of CAB extracts. Furthermore, the CAB extracts strongly decreased the CSH of *S. aureus*. Additionally, FT-IR analysis of *S. aureus* treated with CAB extracts evidenced the reduction in cellular components compared to their respective controls. Thus, the present study reports for the first time, *B. firmus*—a coral-associated bacterium, as a promising source of antibiofilm agent against the recalcitrant biofilms formed by multidrug resistant *S. aureus*.

## 1. Introduction

Biofilms are biotic and abiotic surface-associated, complex and highly structured sessile bacterial communities entrench themselves in a self-produced extracellular matrix of exopolysaccharide (EPS) along with proteins and micro molecule such as DNA [1]. Biofilm-associated bacteria render huge tolerance towards both antibiotics and host immune defence mechanism often resulting in persistent and difficult-to-treat infections, leaving behind their planktonic counterparts [2, 3]. Bacteria that adhere to implanted medical devices or damaged tissue can become dreadful by causing persistent infections through biofilm formation [4].

*Staphylococcus aureus *is one among the biofilm forming human pathogens that causes an array of diseases, ranging from minor localized skin lesions to life-threatening deep tissue damage, and systemic infections such as pneumonia, endocarditis, and exotoxin syndromes [5]. The chronic infections persist and cause significant morbidity and mortality in human population due to the development of recalcitrant biofilm structures by this pathogen. Though *S. aureus* have shown increased *in vitro* antimicrobial susceptibility against chemotherapeutics, the therapy used to treat infections by them often ends up unsuccessful and results in recurrent clinical and chronic subclinical infections. These recurrent and chronic staphylococcal infections can be attributed to the growth of bacteria as biofilms [6, 7].

This notorious biofilm architecture of *S. aureus* composed of an extracellular matrix encases proteins, DNA, and/or polysaccharide glycocalyx (also called the polysaccharide intercellular adhesin or PIA) [8]. Its adhesion to medical devices is incriminated in their pathogenesis. The ability of *S. aureus* to cause diverse diseases has been linked to the numerous virulence factors such as adhesins and toxins [9]. Biofilm formation by *S. aureus* can be governed in part by the production of PIA. PIA plays a key role in subsequent cell-to-cell interactions or quorum sensing (QS), which has been synthesized by icaADBC-encoded proteins [10, 11]. The accessory gene regulator (*agr*) locus, a well-characterized two-component regulatory system, plays a critical role in the regulation of exotoxin production by *S. aureus* [12].

Conventionally, antibiotics used to treat these biofilm-forming pathogens are not targeted against the recalcitrant biofilms; rather they target their planktonic counterparts, which create selective pressure on bacteria and gets resistance against the particular drug [13]. This leads to the stunning increase in the methicillin-resistant *S. aureus*, in addition to the emergence of vancomycin-resistant and to other antibiotics, which hastened and widened greater attention for alternative strategies in development of new therapeutic agents to aid in the prevention and treatment of life-threatening infections caused by these multidrug-resistant (MDR) Staphylococci. Though several naturally produced plant molecules such as terpenoids, glycosteroids, flavonoids, and polyphenols are receiving sustained attention because of their potent antimicrobial, anti-inflammatory, and antitumour properties against pathogenic bacteria, particularly Gram-positive organisms [14], they were not yet reported to exhibit antibiofilm activity against them. Unlike these plant-derived compounds, many marine bacteria were well documented recently for their effective antibiofilm property against both Gram-positive and Gram-negative bacterial pathogens [15–17]. 

Coral reefs are among the most productive marine ecosystems and still remain an untapped resource with a large group of structurally unique biosynthetic products holding enormous biotechnological applications in the form of microorganisms. The probability of finding novel bioactive molecules from these coral ecosystems is highly promising, since microbial consortia in this ecosystem have not been well characterized. The bacteria as well as actinomycetes isolated from coral ecosystem were being equally proved to play a defensive role by producing secondary metabolites that effectively control the bacterial biofilms [17].

Insight to this context, the current study was focused to investigate the effect of CAB extracts on biofilm formation and virulence production by MDR clinical isolates of *S. aureus*. Additionally, the modifications of various cellular components in test strains upon treatment with CAB extracts were examined by Fourier Transform Infrared spectroscopy (FT-IR). To the best of authors' knowledge, this is the first study ever on *B. firmus*, a coral-associated bacterium for its antibiofilm and antipathogenic potentials against *S. aureus*, in particular MDR methicillin-resistant and -susceptible *S. aureus *(MSSA).

## 2. Materials and Methods

### 2.1. Bacterial Strains and CAB Extract Preparation

The clinical strains were isolated from pharyngitis patients attending the Thoracic Science Department at the Rajaji Government Hospital, Madurai. Five clinical strains of methicillin-resistant *Staphylococcus aureus* (MRSA-20, MRSA-44, MRSA-45 MRSA-395 and MRSA-410) and five clinical strains of methicillin-susceptible *Staphylococcus aureus* (MSSA-A8, MSSA-46, MSSA-51, MSSA-84, and MSSA-79) were used in the present study. The ten clinical isolates were identified at species level based on 16S rRNA gene sequencing and their GenBank accession numbers are JN315147, JN315148, JN315149, JN390832, and JN315150 for MRSA and JN315152, JN315153, JN315154, JN390831, and JN315151 for MSSA isolates, respectively. Two *S. aureus* strains, MRSA ATCC 33591 and ATCC 11632 were used as reference strains. 

*S. aureus* strains were grown and maintained on Tryptic soy agar/broth (TSA/TSB) and CAB were cultured on Zobell marine agar/broth (ZMA/ZMB) plates at room temperature. CAB were isolated from the mucus and tissue of the coral *Acropora digitifera* at Gulf of Mannar [15]. Nine CAB were assessed for their abilities to inhibit biofilms of MRSA and MSSA strains, and extracts of CAB-E2 and CAB-E4 which showed excellent inhibition were prepared as described previously [16]. The isolates CAB-E2 (GenBank accession number EU660357) and CAB-E4 (EU660363) were identified as *Bacillus firmus* and *Vibrio parahemolyticus* respectively through 16S rRNA gene sequencing.

### 2.2. Biofilm Formation Assay in 24-Well Polystyrene Plate

The effect of CAB extracts on biofilm formation was done in 24-well polystyrene plates. Briefly, overnight cultures of the test organisms (1%) were inoculated with 1 mL of fresh TSB in the presence (treated) and absence (untreated) of CAB extracts (10–150 *μ*g/mL). The plates were incubated for 24 h at 37°C. After incubation, the plates were washed with sterile phosphate buffered saline (PBS) to remove the planktonic cells and allowed to air dry before being stained. The biofilms were stained with 0.4% crystal violet solution (w/v) for 5 min. Subsequently the unstained dye was discarded, and the wells were rinsed twice with deionized water and then allowed to dry. Finally, 1 mL of absolute ethanol was added in each well. The optical density was determined at 570 nm [16], and percentage of biofilm inhibition was calculated using the following formula:

(1) Percentage  of  inhibition =[(Control  OD570 nm−Test  OD570 nm) /Control  OD570 nm]×100.

The biofilm inhibitory concentration (BIC) was determined as the lowest concentration that produced visible disruption of biofilm formation and a significant reduction in the readings when compared with that of the control wells at OD570 nm. Wells containing medium and extract were used as blanks. Thus, the BIC was determined by both spectrophotometric quantification and also by microscopic visualization, and all the following assays were done at the determined BIC of the CAB extracts E2 and E4.

### 2.3. Antibacterial Activity by Disc Diffusion Assay

In order to check whether the CAB extracts E2 and E4 have any antimicrobial efficacy against MRSA and MSSA isolates, agar well diffusion assay was performed using Muller-Hinton agar (MHA) (Himedia) (Clinical and Laboratory Standards Institute, 2006). Overnight cultures of MRSA and MSSA isolates were subcultured in TSB until a turbidity of 0.5 McFarland (1 × 10^8^ CFU/mL) were attained. Using a sterile cotton swab, the cultures were uniformly spread over the surface of the agar plate. Absorption of excess moisture was allowed to occur for 10 min. In the swabbed plate, wells were punched with a diameter of 10 mm and loaded with extract (at the concentration of 100 *μ*g/mL). One hundred *μ*L of CAB culture supernatant was added in each well. The MHA plates were then incubated at 37°C, and the plates were observed for zone of inhibition after 24 h. 

### 2.4. Effect of CAB Extracts on Growth

The effect of CAB extracts on cell density was done in 24-well polystyrene plates. Wells with TSB were inoculated with clinical strains of MRSA and MSSA isolates along with reference strains, respectively, and treated with two CAB extracts E2 and E4 and incubated at 37°C for 24 h. Wells without extracts acted as controls for respective strains and TSB with extract served as the blank. After 24 h, the contents of the wells were tapped gently and quantified spectrophotometrically for changes in growth at OD600 nm.

### 2.5. In Situ Visualization of *S. aureus*

#### 2.5.1. Light Microscopic Analysis

For visualization by light microscopy, the biofilms were allowed to grow on glass pieces (1 × 1 cm) placed in 24-well polystyrene plates supplemented with and without CAB extracts (10–150 *μ*g/mL) and incubated for 24 h at 37°C. The glass pieces were then washed with PBS and stained with 0.4% crystal violet. The stained glass pieces were placed on slides with the biofilm directed upwards and inspected with a light microscope (Euromex, model: GE3045, the Netherlands) at a magnification of ×400. Visible biofilms were documented with an attached digital camera (Cmex camera, model: DC5000, the Netherlands) [16]. 

#### 2.5.2. Confocal Laser Scanning Microscopic (CLSM) Analysis

The effect of two CAB extracts (10–150 *μ*g/mL) on biofilm formation was monitored under a confocal laser scanning microscope (CLSM) (model: LSM 710) (Carl Zeiss, Germany) after washing with PBS and staining with 0.1% acridine orange. The 488 nm argon laser and a 500–640 nm band pass emission filter were used to excite and detect the stained cells. CLSM images (*N* = 20) were obtained from the triplicate of 24 h old control and treated biofilms and processed using Zen 2009 image software [18]. The Z-stack analysis (surface topography and three-dimensional architecture) was done with the Zen 2009 software (Carl Zeiss, Germany). Further, the images (biofilm stack) were analyzed using COMSTAT software (kindly gifted by Dr. Claus Sternberg, DTU Systems Biology, Technical University of Denmark). Three different parameters that is an average and maximum thickness (*μ*m) of the biofilms and the biovolume (*μ*m^3^), which is the volume of bacteria per *μ*m^2^ of glass surface used were obtained. 

### 2.6. Phenotypic Detection of Slime Production by Congo Red Agar (CRA) Plate Assay

Test and reference strains were screened for the qualitative slime production by CRA plate assay as described by previous method [19]. The CRA medium is composed of TSB (30 gms/L), sucrose (36 gms/L), agar powder (18 gms/L), and Congo red dye (0.8 gms/L). Congo red stain was prepared as a concentrated aqueous solution, autoclaved separately and added to the media when the agar had cooled to 55°C. Plates were inoculated and incubated aerobically for 24 h at 37°C. Biofilm-positive strains produced black-coloured colonies, whilst biofilm-negative strains were coloured pink. The CRA assay was also used to evaluate directly the effect of extracts CAB-E2 and CAB-E4 on slime production. Bacterial extracts at their BIC were mixed together (aseptically) with Congo red and added to the media when the agar had cooled to 55°C. Control plates containing Congo red stain without CAB extracts were also prepared. Plates were then inoculated with test strains and were incubated aerobically for 24 h at 37°C.

### 2.7. Bacterial Adhesion to Hydrophobicity (BATH) Assay

The effect of CAB extracts E2 and E4 on the CSH of the test strains was determined by using MATH (microbial adhesion to hydrocarbons) assay as a measure of their adherence to the hydrophobic hydrocarbon (toluene) following the procedure described by [20]. Briefly, 1 mL of test bacterial culture (OD530 nm = 1.0) was placed into glass tubes and 100 *μ*L of toluene along with the extracts at BIC was added. The mixtures were vigorously vortexed for 2 min and incubated for 10 min at room temperature to allow phase separation, and then the OD530 nm of the aqueous phase was recorded. Bacterial culture incubated with toluene devoid of extracts acted as controls. The percentage of hydrophobicity was calculated according to the formula: % hydrophobicity = [1 − (OD530 nm after vortexing/OD530 nm before vortexing)] × 100.

### 2.8. EPS Quantification Assay

The total carbohydrate assay was done for the determination of EPS. Briefly, sterile glass slides were immersed in test culture with CAB extract at their BIC in 24-well polystyrene plates and incubated for 24 h. After incubation, the glass slides were removed and washed with 0.9% NaCl. The cell suspensions in 0.9% NaCl were transferred to test tubes with an equal volume of 5% phenol to which 5 volumes of concentrated H_2_SO_4_ containing 0.2% of hydrazine sulphate had been added. The mixture was incubated in dark for 1 h, and the absorbance was measured at OD490 nm after centrifugation (10,000 g for 10 min) [21].

### 2.9. Hemolysin Assay

The detection of hemolytic activity of control and treated samples were primarily done by using blood agar plates, prepared by adding 5% washed erythrocyte to nutrient agar. Total hemolysis assay was carried out as described previously [22]. In brief, 1% inoculum of overnight test cultures added with CAB extracts at BIC were grown in Muller Hinton broth (MHB) supplemented with 0.5% Tween 80 and incubated at 37°C until it reaches OD600 nm of 2.5. After centrifugation, 100 *μ*L of cell-free supernatants were incubated at 37°C for 30 min along with 900 *μ*L of a suspension containing 2% sheep erythrocytes, 20 mM CaCl_2_, 10 mM Tris, and 160 mM NaCl adjusted to pH 7.4 with hydrochloric acid. Then, the mixture was kept on ice for 20 min and centrifuged. The released hemoglobin in the supernatant was determined at OD530 nm. The results were indicated as the percentage lysis compared with the erythrocytes lysed in distilled water.

### 2.10. FT-IR Analysis

Two CAB extracts at their BIC were treated with the cell suspension (10^6^ CFU/mL) of each strain from MRSA and MSSA along with control samples devoid of extracts. After incubation at 37°C for 24 h at 160 rpm, the cell suspension was centrifuged at 10000 rpm at 4°C for 10 min., and the supernatants were discarded. The FT-IR spectra in the 400–4000 cm^−1^ region were recorded on a Tensor 27 FT-IR Spectrometer equipped with DLaTGS detector (Bruker). The frequencies for all sharp bands were determined accurately from the original baseline-corrected spectra belonging to the corresponding groups. The spectra were obtained using potassium bromide (KBr) pellet technique. Potassium bromide (AR grade) was dried under vacuum at 100°C for 48 h, and 100 mg of KBr with 1 mg of bacterial culture pellet was taken to prepare KBr pellet. For each spectrum, 64 scans were collected at a resolution of 4 cm^−1^. The spectra were plotted as absorbance versus number, and principal components (PCs) analysis was performed using portable Unscrambler version 9.7.

### 2.11. Statistics

Statistical analysis was performed using SPSS. Values were expressed as mean ± SD. A Duncan's ANOVA test was used to compare parameters between groups and a Dunnett's ANOVA test to compare between tests and control.

## 3. Results 

### 3.1. Assessment of MRSA and MSSA Biofilm Formation

A total of 102 MSSA and 63 MRSA clinical strains were screened for the formation of biofilm (data not shown), out of which, five potent biofilm forming strains each from MRSA and MSSA were chosen for further study. 

Qualitatively, biofilm formation of five MRSA (MRSA-20, MRSA-44, MRSA-45 MRSA-395, and MRSA-410) and five MSSA clinical isolates (MSSA-A8, MSSA-46, MSSA-51, MSSA-84, and MSSA-79) were analyzed through light microscopic observations at the magnifications ×400 and quantitatively analyzed by spectrophotometry at OD570 nm.

### 3.2. Determination of BIC

Out of 9 CAB screened for antibiofilm activity (data not shown), two extracts, namely CAB-E2 (*Bacillus firmus*) and CAB-E4 (*Vibrio parahaemolyticus*) shown excellent inhibition against the biofilms of both MRSA and MSSA strains. The ethyl acetate extracts of CAB-E2 and CAB-E4 were weighed, and their yields were found to be 0.016% (160 *μ*g/mL) and 0.011% (110 *μ*g/mL), respectively. To determine the BIC of these 2 CAB extracts on *S. aureus*, extracts with varied ranges of concentrations (10–150 *μ*g/mL) were used. Concentration-dependent decrease in biofilm formation of test pathogens was obtained upon treatment with the extracts E2 and E4. These two extracts showed a prominent biofilm inhibition of 80–85% at a concentration of 100 *μ*g/mL. CAB-E2 inhibited the biofilm formation of MRSA and MSSA strains up to 83 and 87%, respectively, at a concentration 100 *μ*g/mL (Figures 5(a) and 5(b)), whereas extract E4 has shown a maximum biofilm inhibition of 79 and 84% against MRSA and MSSA isolates (Figures 5(c) and 5(d)) at the same concentration. Hence, 100 *μ*g/mL was fixed as the BIC for both the extracts, and further assays were done at this concentration of extracts.

### 3.3. Effect of Extracts on *S. aureus* Growth

The CAB extracts were evaluated at their BICs (100 *μ*g/mL) for their antibacterial activity by agar disc diffusion method. There was no antibacterial activity as there was no zone of inhibition around the loaded wells (Figures 6(A) and 6(B)). This was further confirmed by spectrophotometric analysis at OD600 nm, as there was no bactericidal or bacteriostatic activity observed (Figures 7(a) and 7(b)). It was obvious that the growth of *S. aureus* was the same even in the presence of extracts at their BIC as that of the control samples.

### 3.4. In Situ Analysis of Biofilm Formation

**Figure 1:**
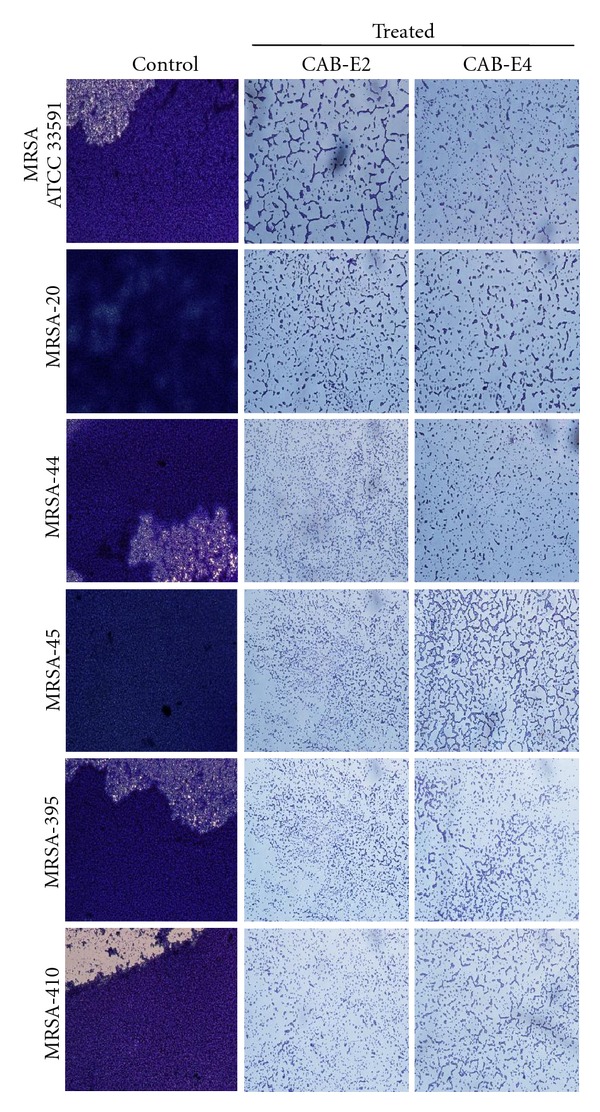
Light microscopic images (×400) revealing the antibiofilm activity of the extracts CAB-E2 and E4 at their BICs (100 *μ*g/mL) against MRSA isolates.

**Figure 2:**
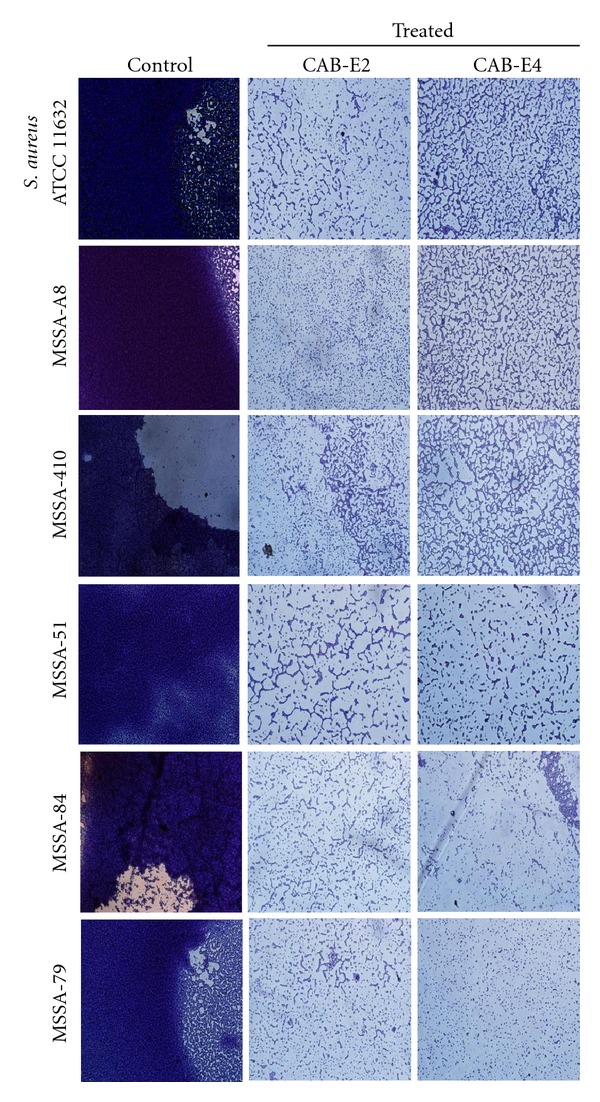
Light microscopic images (×400) revealing the antibiofilm ability of CAB extracts E2 and E4 at their BICs (100 *μ*g/mL) against MSSA isolates.

**Figure 3:**
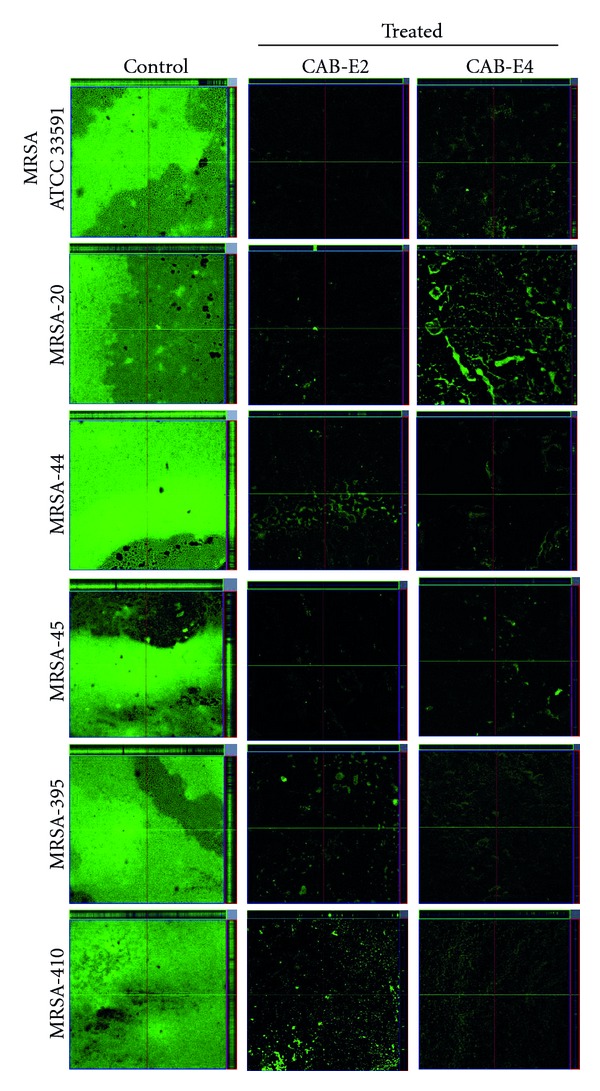
CLSM images demonstrating the antibiofilm potentials of CAB-E2 and E4 against MRSA isolates at their BIC of 100 *μ*g/mL.

**Figure 4:**
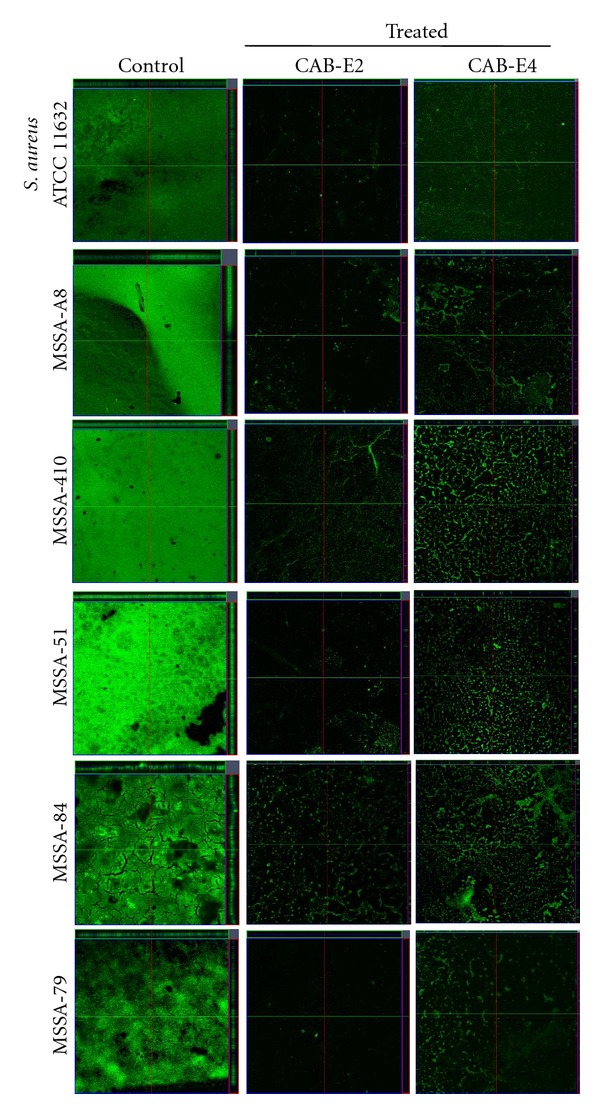
CLSM images demonstrating the antibiofilm potentials of CAB-E2 and E4 against MSSA isolates at their BIC of 100 *μ*g/mL.

**Figure 5:**
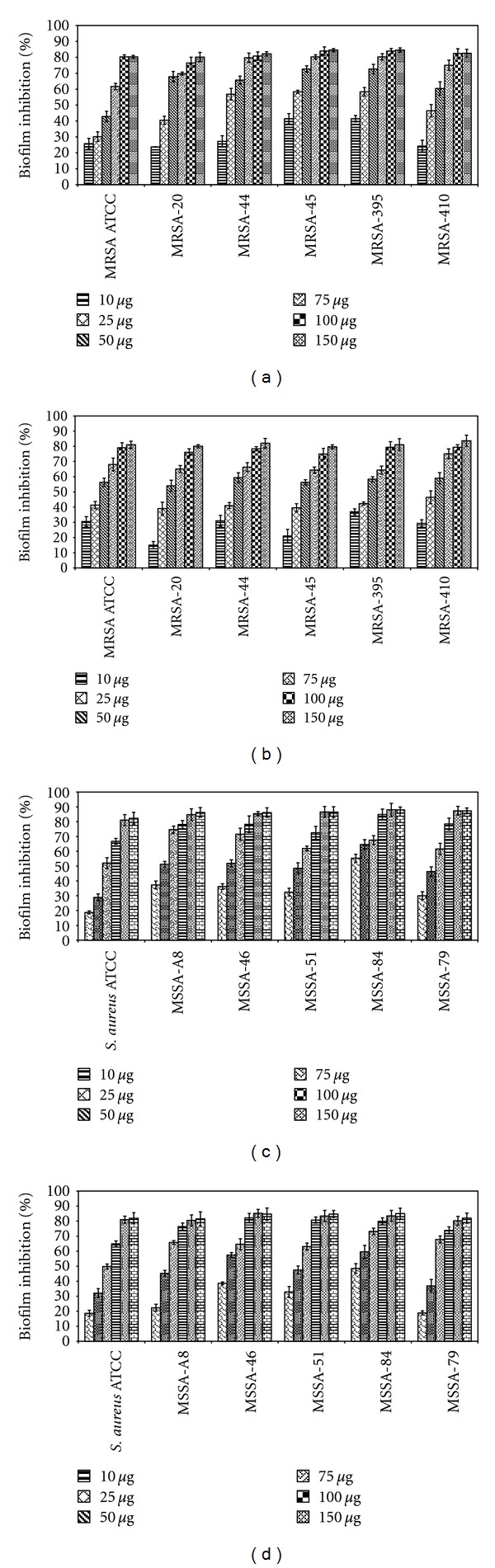
Effect of CAB-E2 and E4 on biofilm formation of MRSA and MSSA isolates along with the reference strains; antibiofilm activity of CAB-E2 against (a) MRSA and (b) MSSA isolates; CAB-E4 against (c) MRSA and (d) MSSA isolates, quantified by crystal violet adsorption and measuring absorbance at 570 nm. CAB extracts of varied concentrations ranging from 10 to 150 *μ*g/mL were used in order to probe the BIC. 100 *μ*g/mL was fixed as the BIC for both the extracts, as this concentration shows maximum inhibition of biofilm. Mean values of triplicate individual experiments and SDs are shown.

**Figure 6:**
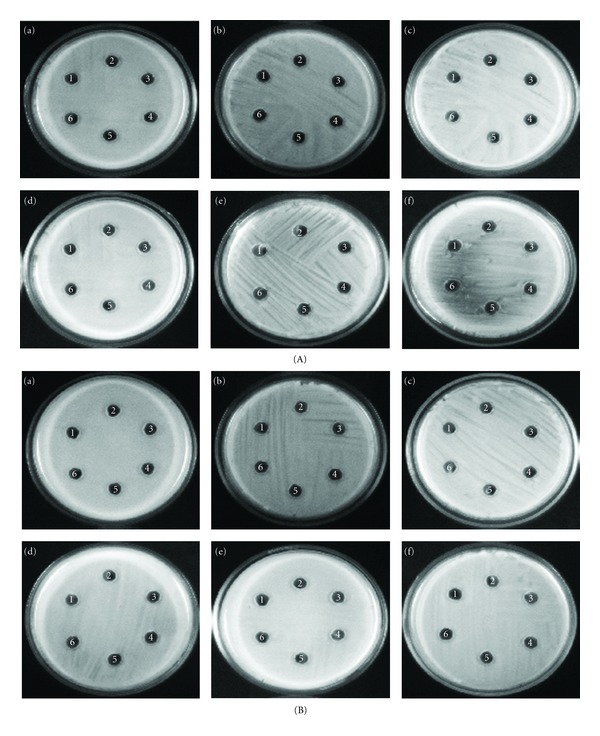
(A) Effect of CAB-E2 and E4 on the growth of MRSA isolates and reference strain. Antibacterial activity of CAB extracts on (a) MRSA ATCC 33591, (b) MRSA-20, (c) MRSA-44, (d) MRSA-45, (e) MRSA-395, and (f) MRSA-410. Wells 1, 2, 3, 4, and, 5 contained CAB-E2 (100 *μ*g), CAB-E4 (100 *μ*g), cell-free culture supernatant of CAB-E2 (100 *μ*L), cell-free culture supernatant of CAB-E4 (100 *μ*L), ethyl acetate (100 *μ*L) respectively, and well 6 served as the negative control in all plates. (B) Effect of CAB-E2 and E4 on the growth of MSSA isolates and reference strain. Antibacterial activity of CAB extracts on (a) MSSA ATCC 11632, (b) MSSA-A8, (c) MSSA-46, (d) MSSA-51, (e) MSSA-84 and, (f) MSSA-79. Wells 1, 2, 3, 4, and 5 contained CAB-E2 (100 *μ*g), CAB-E4 (100 *μ*g), cell-free culture supernatant of CAB-E2 (100 *μ*L), cell-free culture supernatant of CAB-E4 (100 *μ*L), ethyl acetate (100 *μ*L) respectively, and well 6 served as the negative control in all plates.

**Figure 7:**
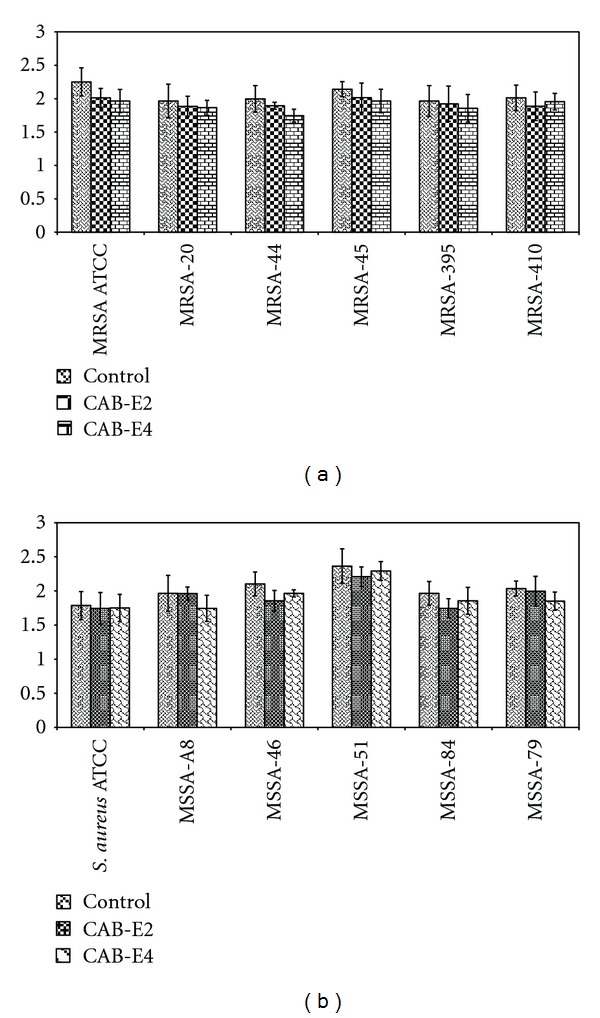
Effect of CAB-E2 and E4 on the growth of MRSA (a) and MSSA (b) isolates at 100 *μ*g/mL (BIC), samples without extracts act as the control for respective strains.

The images obtained from light microscopic analysis revealed that the control slides depicted well developed biofilm formation of test pathogens, whereas, the test pathogens upon treatment with CAB extracts (100 *μ*g/mL) developed poor biofilm formation compared to that of control sample (Figures 1 and 2). To ascertain the results obtained in light microscopy (i.e., disintegration of biofilm structures by CAB extracts), we used CLSM to further elucidate the antibiofilm potential of extracts against biofilms of MDR *S. aureus* strains (Figures 3 and 4). CLSM images unveiled the strong adhering ability of reference strain MRSA ATCC 33591, which lead to the development of dense biofilm formation on glass piece of control samples, while treated samples depicted the antibiofilm potential of CAB-E2 and E4 by disintegrating the recalcitrant biofilm architecture of reference strain upon treatment. The significant reduction in different parameters of MRSA and MSSA biofilms such as maximum thickness (*μ*m), biovolume (*μ*m^3^), and average thickness (*μ*m), upon treatment with CAB extracts, was evidenced through COMSTAT analysis (Tables 1(a) and 1(b)).

### 3.5. Inhibition of Slime Synthesis (CRA)

**Figure 8:**
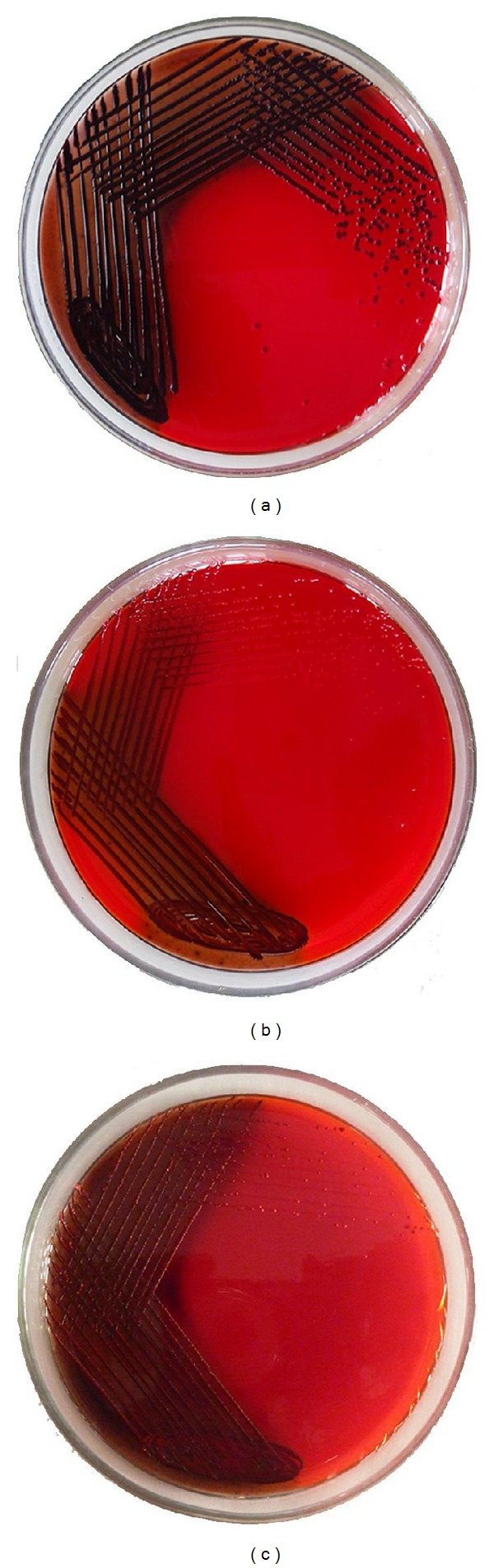
Bacterial colonies of clinical strains MRSA-20 grown on Congo red agar plates, showing decreasing levels of slime production in the presence of CAB extracts at BIC (100 *μ*g/mL): (a) strong black colour colonies of untreated MRSA-20; (b) MRSA-20 treated with CAB-E2; (c) MRSA-20 treated with CAB-E4 at BIC.

As a prelude to assess the inhibition of biofilm synthesis of methicillin-resistant and-susceptible *S. aureus* strains, the ability of CAB extracts to inhibit the synthesis of slime was qualitatively examined on CRA plate assay. CAB extracts at their BICs were incorporated into CRA plates to substantiate whether the growing colonies could show any change in colour from black to red or Bordeaux red. Both the CAB-E2 and E4 were potent enough to inhibit the slime production of almost all the test strains along with the reference strains and because of the similarity in the pattern of results obtained, only the results from representative MRSA and MSSA isolates are shown (Figure 8). 

### 3.6. Effect of CAB Extracts on EPS

**Figure 9:**
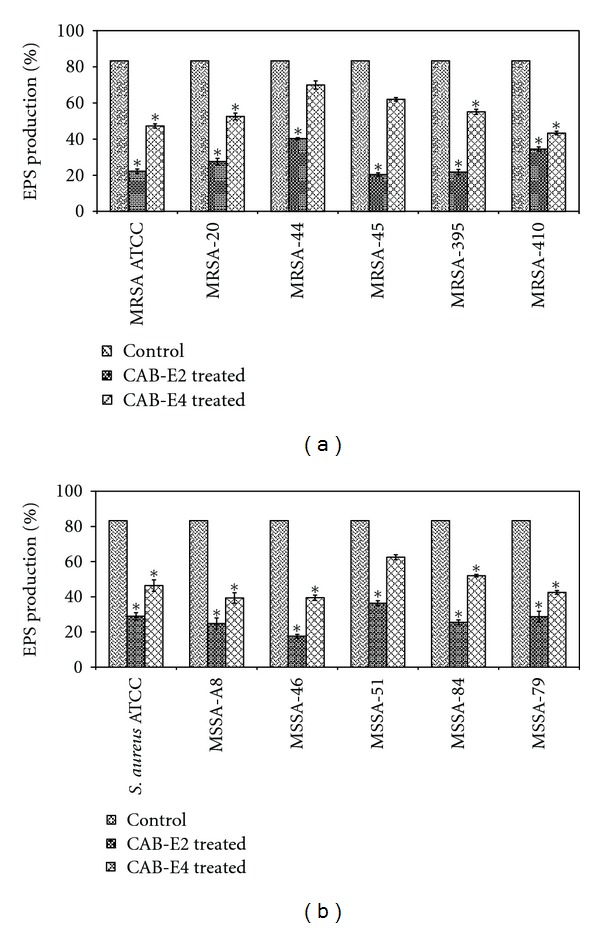
Effect of CAB-E2 and E4 on the EPS production of MRSA (a) and MSSA (b) isolates at a concentration of 100 *μ*g/mL (BIC). Mean values of triplicate individual experiments and SDs are shown. *indicate the statistical significance (*P* < 0.05).

The spectroscopic analysis of EPS extracted from control and treated samples revealed that the extracts E2 and E4 had significantly reduced the synthesis of EPS in both MRSA and MSSA isolates. Interestingly, extract E2 showed a maximum reduction of 76 and 79% in MSSA and MRSA isolates, respectively, (Figures 9(a) and 9(b)). Whereas, extract E4 depicted comparatively low percentage of EPS reduction with that of CAB-E2. 

### 3.7. Effect of CAB Extracts on Extracellular Hemolysin Production

While exoproteins are mainly secreted during post-exponential growth, the test pathogens were cultured with CAB extracts at their BICs to an OD600 nm of 2.5. A statistically significant reduction in hemolysin was observed in both MRSA and MSSA isolates treated with the extracts, particularly CAB-E2 (Tables 2(a) and 2(b)).

### 3.8. Effect of CAB Extracts on CSH (MATH Assay)

Cell surface hydrophobicity (CSH) plays a vital role for the adhesion property of biofilm-producing bacteria. The percentage hydrophobicity was high up to 62, 51 and 44% in isolates of MRSA-20, MRSA ATCC, and MRSA-45, respectively, whereas it was lesser than 40% for the two remaining MRSA isolates. In contrast, MSSA isolates showed relatively lesser hydrophobicity of 33–20% compared to that of MRSA isolates. In order to check the ability of CAB extracts to uphold changes in the cell surface properties of the test pathogens, they were exposed in toluene with the two CAB extracts. The extracts were tested at their BICs corresponding to each pathogen tested. CAB-E2 was able to reduce the hydrophobic properties of the MRSA and MSSA cell membrane, decreasing by at least onefold of its hydrophobic nature compared with that of untreated controls. (Tables 3(a) and 3(b)). However, CAB-E4 did not display any recognizable modification on the cell hydrophobicity of the test pathogens (Tables 3(a) and 3(b)).

### 3.9. FT-IR Analysis

**Figure 10:**
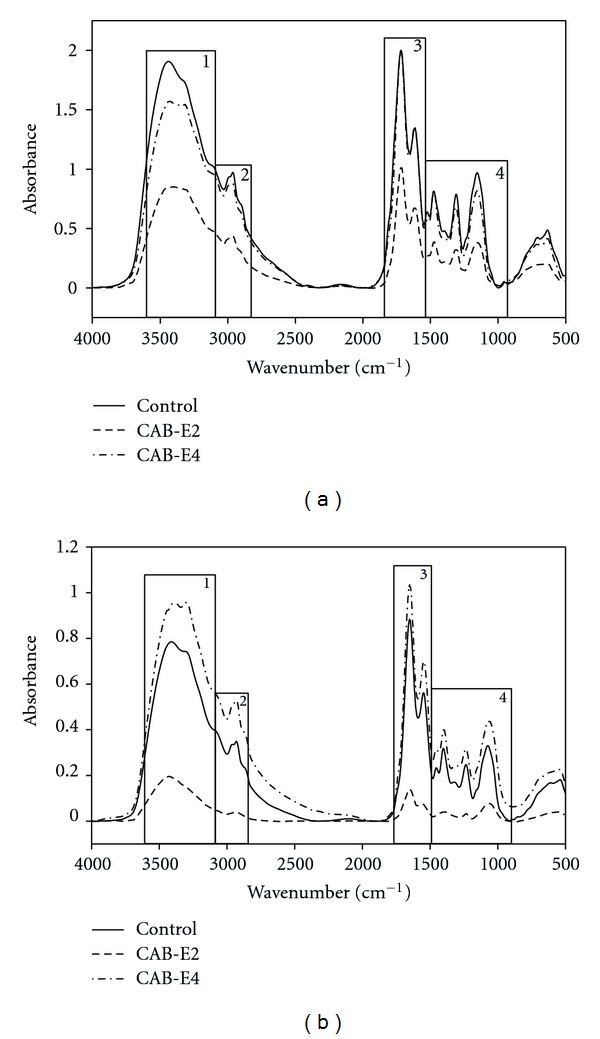
FT-IR spectra of MRSA-44 (a) and MSSA-A8 (b) treated with CAB-E2 and E4. The regions showing maximum variation were taken for analysis: (1) 3500–3100 cm^−1^: hydration of bacterial cells; (2) 3050–2700 cm^−1^: fatty acids in the bacterial cell membrane; (3) 1700–1600 cm^−1^: amide linkage from proteins and peptides; (4) 1500–1000 cm^−1^: mixed region, proteins, and fatty acids.

The FT-IR spectra depicted visual variations between the control cells and cells treated with CAB extracts. In the FT-IR spectrum, CAB-treated MRSA and MSSA cells show maximum absorbance at the four most predominant regions such as 3700–3100 cm^−1^: hydration of bacterial cells; 3050–2750 cm^−1^: fatty acids in bacterial cell membrane; 1700–1600 cm^−1^: amide linkage from proteins and peptides; 1500–1000 cm^−1^: mixed region, proteins, and fatty acids (Figures 10(a) and 10(b)) [23].

**Figure 11:**
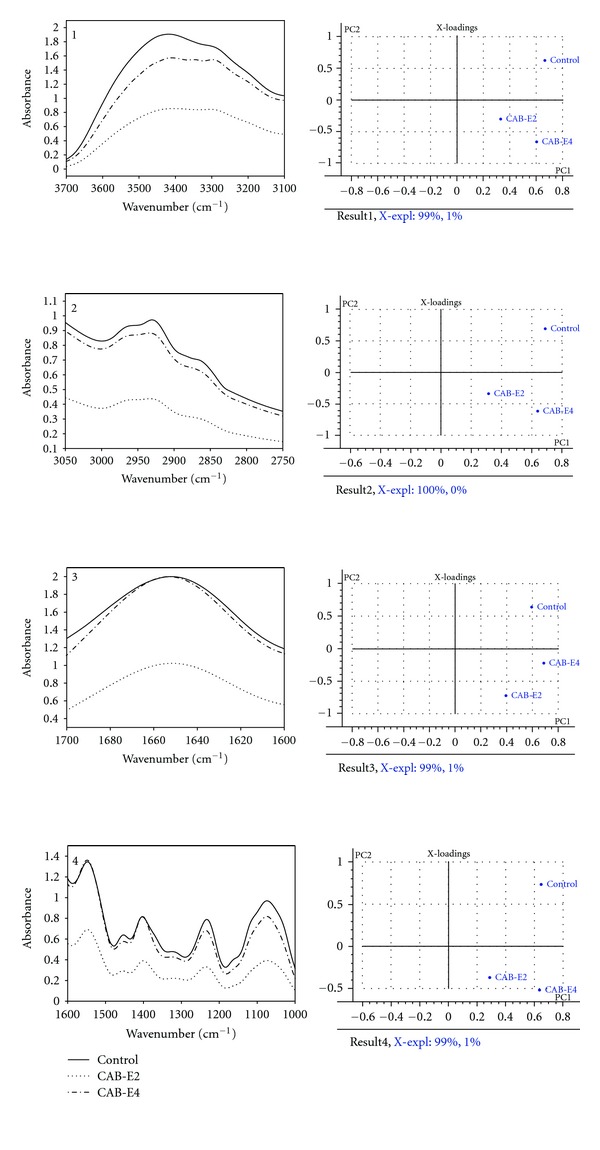
FT-IR spectra and principal component analysis of MRSA-44 treated with CAB-E2 and E4. (1) 3500–3100 cm^−1^: hydration of bacterial cells; The explained variation in *X* by PC1 and PC2 was 99 and 1%, respectively. (2) 3050–2700 cm^−1^: fatty acids in the bacterial cell membrane; The explained variation in *X* by PC1 and PC2 was 100 and 0%, respectively. (3) 1700–1600 cm^−1^: amide linkage from proteins and peptides; The explained variation in *X* by PC1 and PC2 was 99 and 1%, respectively. (4) 1500–1000 cm^−1^: mixed region, proteins, and fatty acids; The explained variation in *X* by PC1 and PC2 was 99 and 1%, respectively.

**Figure 12:**
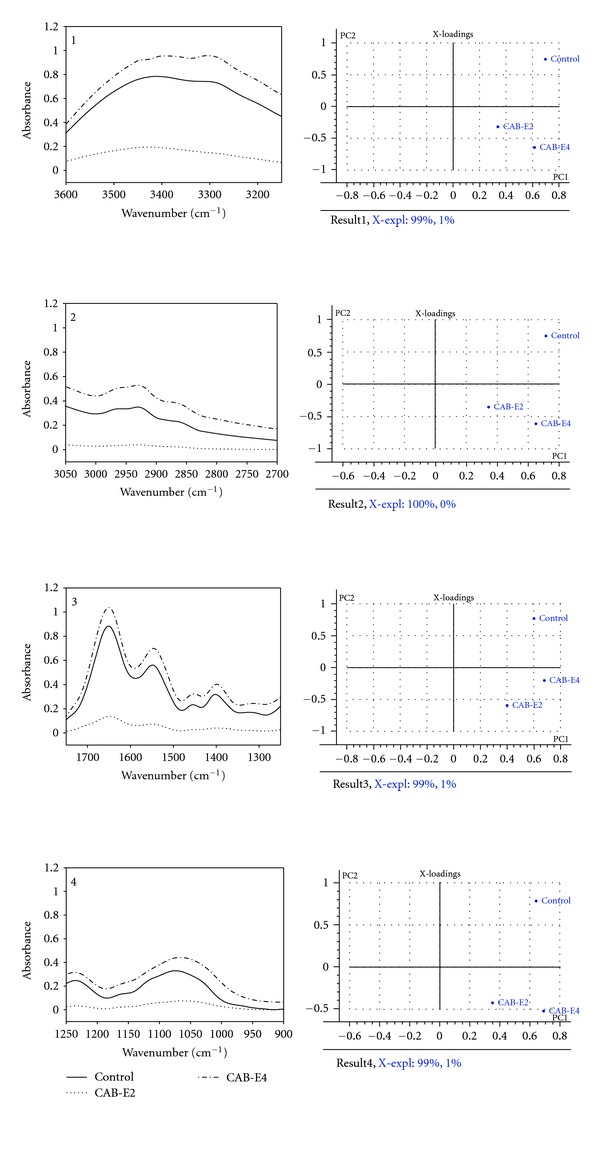
FT-IR spectra and principal component analysis of MSSA-A8 treated with CAB-E2 and E4. (1) 3500–3100 cm^−1^: hydration of bacterial cells; The explained variation in *X* by PC1 and PC2 was 99 and 1%, respectively. (2) 3050–2700 cm^−1^: fatty acids in the bacterial cell membrane; The explained variation in *X* by PC1 and PC2 was 100 and 0%, respectively. (3) 1700–1600 cm^−1^: amide linkage from proteins and peptides; The explained variation in *X* by PC1 and PC2 was 99 and 1%, respectively. (4) 1500–1000 cm^−1^: mixed region, proteins, and fatty acids; The explained variation in *X* by PC1 and PC2 was 99 and 1%, respectively.

The CAB-E4 treated MSSA cells showed enhanced hydration of cells, whereas dehydration was observed with CAB-E2-treated MRSA and MSSA cells. There were vast differences in the fatty acid profile region of control and extract-treated cells. In particular, *S. aureus* cells treated with CAB-E2 showed declined peak of absorbance at the fatty acid region, while CAB-E4 made some reduced modifications in MRSA. Contrary to expectation, MSSA cells treated with CAB-E4 showed elevated peak of absorbance at the fatty acid region compared to that of control. The peak in the 1650 cm^−1^ implies the interactions between the proteins and peptides [24]. Though there was not much variation upon treatment with CAB-E4 it was significant in both MRSA cells treated with CAB-E2 at the protein region. Whereas CAB-E4-treated MSSA cells showed inclined peak of absorbance with of control. The peak found in the range of 1500–1200 cm^−1^ shows the interaction of protein and fatty acids [23]. Compared to CAB-E4, MRSA and MSSA cells treated with CAB-E2 showed a decreased fatty acid secretion than that of control. The peaks found in the range of 1200–900 cm^−1^ which show the symmetric phosphate bond stretching in the nucleic acids and polysaccharides within the cells [25]. In the range of 1200–900 cm^−1^, CAB-E2-treated MRSA and MSSA cells show huge reduction in the polysaccharides, where as CAB-E4 did not show even marginal changes compared to that of control. The PC analysis also demonstrated substantial variations in the CAB extracts treated and untreated *S. aureus* cells (control). In the entire analyzed spectral region, the control and the CAB extracts-treated cells almost lie in different quadrants (Figures 11 and 12).

## 4. Discussion

Bacteria from marine environment are known for their rich source of bioactive molecules particularly for plenty of antibiotic compounds against various Gram-positive and Gram-negative pathogens. Nevertheless, reports are scanty for their antibiofilm potentials [16–18]. Moreover, several studies suggest independently that many marine bacteria are capable of producing novel antibiofilm compound(s) which have not been tapped from terrestrial environment [26, 27]. The coral ecosystem remains an unexplored resource with surplus pharmaceutically potent bacteria, especially for their antibiofilm activity against MDR *S. aureus. *Hence, in the present study, we investigated the biofilm-inhibiting properties followed by sequential virulence inhibition potentials of two coral-associated bacterial extracts against the clinical isolates of MRSA and MSSA.

 Bacterial growth occurs rapidly soon after its attachment to a solid substratum, which is the foremost stage in biofilm formation. For initial few hours of growth on the surface, the adhesion is reversible [28, 29]. Therefore, preventing the bacterial adhesion at the very initial stage can considerably reduce the risk of further biofilm formation. The active metabolites of CAB extracts prominently inhibited the biofilm formation at its early stage through reducing the microcolonies formed by *S. aureus*. CAB extracts reduced biofilm formation up to 85 and 87% at a concentration of 100 *μ*g/mL against MRSA and MSSA isolates, respectively. The 24-well polystyrene plate assay is the most extensively used assay for the detection of biofilm formation [30]. Both the biofilm inhibition assay and microscopic observations clearly portrayed that the marine bacterial extracts CAB-E2 and CAB-E4 effectively reduced and dispersed the microcolonies. 

Next major stage in biofilm development is the formation of the characteristic biofilm architecture [31]. From the microscopic images, it is exceptionally appropriate to state that CAB extracts imposed a maximum collapse on the recalcitrant biofilm architectures of MDR-MRSA and MSSA clinical isolates by loosening the microcolonies. It is envisaged that the natural products of CAB possibly interfered at any step of the Gram-positive pathogens' biofilms but evidently did not inhibit the growth of bacteria at all BICs [32]. The architecture of biofilms formed by all test isolates observed through CLSM further validated the antibiofilm efficiency of CAB extracts E2 and E4. The substantial reduction in different parameters of recalcitrant biofilm architecture such as maximum thickness (*μ*m), biovolume (*μ*m^3^), and average thickness (*μ*m), upon treatment with CAB extracts was evidenced through COMSTAT analysis. Effective disruption and reduction of microcolonies were apparent through the CLSM images followed COMSTAT analysis (Tables 1(a) and 1(b)).

Among the 9 CAB extracts screened, CAB-E2 (*Bacillus firmus*) and CAB-E4 (*Vibrio parahaemolyticus*) were found to be the potent biofilm inhibitors of *S. aureus*, since they were proficient enough to inhibit the biofilms of MSSA and multidrug-resistant MRSA at eventually low concentration of the extracts (50–100 *μ*g/mL). The result of this study is very much comparable to the previous study wherein a marine actinomycete (CAA-3) reduced biofilm formation in *S. aureus* up to 80% at a concentration of 100 *μ*g/mL [17]. Previous reports suggest that a chemically diverse library of TAGE-triazole conjugates have the ability to inhibit biofilm formation by *S. aureus* [33] and gentamycin with farnesol as an adjuvant therapeutic agent prevents biofilm-related infections in *S. aureus* [34]. Nevertheless, none of these studies have quantified the biofilm inhibition. 

In *S. aureus*, various physiological activities such as biofilm formation, expression of virulence factors are regulated via QS system [35]. Formation of biofilm is the prime factor responsible for drug resistance in many pathogens particularly Gram-positive bacteria. Moreover, biofilm-forming bacteria develop resistance to antibiotics more frequently than other pathogens [36]. The present study was highly focused on the bacterial extracts that had no effect on the growth of test pathogens, rather effectively controlled their virulence factors by acting upon the biofilms formed under QS. In the antimicrobial assay, neither of the CAB extracts exhibited any antimicrobial activity. It has been a well-known fact that extensive exposure of any antibiotic targeted against bacterial pathogen exerts invariable pressure by which it gains reduced susceptibility and finally attains complete resistance against the particular antibiotic. Hence, this study hypothesizes that the probability of developing resistance to CAB-antibiofilm compounds is considerably low, as it possesses no bactericidal or bacteriostatic effect and no harm to the growth of the bacterial pathogens.

A recent report suggests that EPS leads to alterations in biofilm architecture that correlate with an increased resistance of cells to osmotic and oxidative stresses in addition to killing by biocides such as chlorine [37]. Here, we demonstrated the CAB extracts E2 and E4 inhibiting EPS production of both MRSA and MSSA isolates up to a maximum range of 80–85%. The most important cause for methicillin resistance in *S. aureus* is their capsular polysaccharide [38]. Therefore, reducing the synthesis of EPS may make the biofilm more susceptible to antibiotics. Furthermore, qualitative method (CRA for *S. aureus*) also exhibited that bacterial extracts result in excellent reduction in slime synthesized by biofilm-producing MDR *S. aureus*. The classic black color appearance of biofilm forming test strains was highly reduced and changed to pink on CRA plates incorporated with 2 extracts individually. This may also suggest that the two CAB extracts could possibly interfere with the synthesis of extracellular polysaccharides equivalently, thereby disrupting the formation of glycocalyx and reduce the amount of slime that accumulates.

Alpha-hemolysin plays a major role in biofilm formation and also appears to be primarily required for cell-to-cell interactions that is, QS; therefore a mutant allele of *hla *(gene encoding alpha-hemolysin) can initially help in colonizing substratum but never in organizing multicellular macrocolonies [39]. The results of hemolysin assay clearly evidence that CAB could be a promising quorum quenching compound by significantly reducing the production of alpha-hemolysin, which is being controlled by global regulators such as *agr* and *sarA* [40]. 

The microbial cell surface properties like charge and hydrophobicity play a crucial role in bacterium-host cell interactions [41]. Earlier studies reported the interference of plant extracts on the hydrophobicity of Gram-negative bacteria [42] and Gram-positive bacteria [43, 44] and thus inhibiting biofilm formation. Similarly, CAB-E2 at its BIC was able to modify greatly the cell surface properties of both MRSA and MSSA isolates, decreasing by at least onefold its hydrophobic nature compared with that of untreated controls and thereby significantly contributing to antibiofilm activity (Tables 3(a) and 3(b)). Considerable changes in cell hydrophobicity were observed in *S. aureus* treated with CAB-E4. It has recently been reported in a study that there was a significant difference in the cell surface charge between treated and untreated group B streptococci with antibiotics [45]. Bacteria inside biofilms can produce periplasmic glucans which bind to antibiotics, sequestering them in the periplasm and preventing them from the action of antibiotics [46]. Thus, reducing the hydrophobicity will expose the bacterial biofilm to antibiotics and sequentially will ease the eradication of the biofilm.

In addition to these studies, FT-IR technique has been applied for probing the variation in cellular components of *S. aureus* upon treatment with CAB extracts. For the biomolecules, FT-IR is used to probe the structures and the interaction of the biomacromolecules such as DNA, RNA, proteins, carbohydrates, and lipids isolated from tissues and cells [47]. The representative FT-IR spectra of CAB-E2 and E4-treated *S. aureus* cells in response to the biofilm disturbance show an apparent decrease in absorbance of protein (1800–1500 cm^−1^) and polysaccharide (1200–900 cm^−1^) regions. In the recent past, several researchers have also used similar FT-IR technique for probing the variations in the chemical composition in higher plants and algae [48, 49]. The results obtained in FT-IR spectrum of CAB-E2-treated MRSA and MSSA demonstrate that their capability of producing virulence such as hemolytic protein and EPS was highly diminished. These virulence factors are the prime cause for exerting dreadful pathogenesis by means of developing into biofilm architecture, which has been greatly reduced to a significant level. The FT-IR result goes parallel and highly consistent with the results obtained in the quantitative and qualitative assays of EPS and hemolysin production.

## 5. Conclusion 

Antipathogenic therapy (which targets only the biofilms and its associated virulence rather than harming the growth of the pathogen) is an alternative strategy for currently prevailing antimicrobial therapy (which often results in developing resistance to that particular drug) to treat biofilm-forming pathogens. In the same way, present study reveals the antibiofilm and antipathogenic potentials of coral-associated bacteria from Gulf of Mannar. The extract of *B. firmus* can be a promising antibiofilm agent as it demonstrates significant inhibition in terms of biofilm formation, synthesis of slime, EPS and hemolysin, and most prominently its ability to modify the adhesion properties of MDR-MRSA and MSSA. Finally, FT-IR analysis further validated the effect of CAB extract on phenotypic inhibition of test pathogens. Further characterization of lead molecules from this extract may end up with novel bioactive agents which can be targeted to these dreadful biofilm-forming pathogens.

## Figures and Tables

**Table 1a:** (a)

Strain	Maximum thickness (**μ**m)			Average thickness (**μ**m)			Biovolume (**μ**m^3^/**μ**m^2^)		
	Control	CAB-E2	CAB-E4	Control	CAB-E2	CAB-E4	Control	CAB-E2	CAB-E4
MRSA ATCC 33591	48.3	28.7^∗^	33.5^∗^	42	3.8^∗^	10.4^∗^	40	2.1^∗^	6.2^∗^
MRSA-20	54	27.8^∗^	29.3^∗^	37.8	0.25^∗^	0.94^∗^	44.2	0.57^∗^	1.6^∗^
MRSA-44	49	27^∗^	30.4^∗^	37.8	1.2^∗^	1.9^∗^	34	0.6^∗^	1^∗^
MRSA-45	65.6	39.6^∗^	39.2^∗^	56.7	1^∗^	1.2^∗^	42.6	0.5^∗^	0.5^∗^
MRSA-395	59.7	34.6^∗^	35.7^∗^	53.3	0.3^∗^	2.3^∗^	46.3	1.6^∗^	0.2^∗^
MRSA-410	48.3	20.8^∗^	26.5^∗^	42	0.6^∗^	1.4^∗^	40.1	0.3^∗^	5.5^∗^

**Table 1b:** (b)

Strain	Maximum thickness (**μ**m)			Average thickness (**μ**m)			Biovolume (**μ**m^3^/**μ**m^2^)		
	Control	CAB-E2	CAB-E4	Control	CAB-E2	CAB-E4	Control	CAB-E2	CAB-E4
*S. aureus* ATCC 11632	48.3	14.7^∗^	16.5^∗^	42	2.8^∗^	2.4^∗^	40.1	1.4^∗^	1.5^∗^
MSSA-A8	82.7	12.6^∗^	13.9^∗^	67.1	3.4^∗^	4.3^∗^	57.8	2.3^∗^	3.1^∗^
MSSA-46	23.1	12.6^∗^	14^∗^	21.3	4.4^∗^	5.2^∗^	21.5	3.1^∗^	2.9^∗^
MSSA-51	25.2	11^∗^	12.3^∗^	22.7	2.3^∗^	2.1^∗^	2.2	1.5^∗^	1.5^∗^
MSSA-84	30.3	6.9^∗^	11^∗^	21	0.4^∗^	3.2^∗^	11.2	1.8^∗^	0.2^∗^
MSSA-79	21.8	4.1^∗^	10.2^∗^	19.3	0.2^∗^	3.2^∗^	16.5	0.1^∗^	1.5^∗^

**Table 2a:** (a)

*S. aureus* strains	Hemolysis (%) of sheep erythrocytes by CAB extracts		
	Control (0)	CAB-E2 (100 *μ*g/mL)	CAB-E4 (100 *μ*g/mL)
MRSA ATCC 33591	100%	30.3% ± 4.2**	55.1% ± 3.1*
MRSA-20	100%	54.3% ± 3.6*	57.9% ± 2.8*
MRSA-44	100%	45.3% ± 5.1**	47.4% ± 3.6*
MRSA-45	100%	33.1% ± 2.6**	55.6% ± 5.1*
MRSA-395	100%	57.7% ± 4.6*	55.8% ± 4.2*
MRSA-410	100%	53.5% ± 3.3*	52.1% ± 3.9*

Hemolytic activity in untreated MRSA culture supernatant was set as 100%; values represent means ± SD (*n* = 3). *indicates *P* < 0.05 and **indicates *P* < 0.01 compared to the corresponding controls.

**Table 2b:** (b)

*S. aureus* strains	Hemolysis (%) of sheep erythrocytes by CAB extracts		
	Control (0)	CAB-E2 (100 *μ*g/mL)	CAB-E4 (100 *μ*g/mL)
*S. aureus* ATCC 11632	100%	43.1% ± 3.2**	50.9% ± 1.4*
MSSA-A8	100%	51.9% ± 2.8*	55.6% ± 2.4*
MSSA-46	100%	55.3% ± 1.8*	52.4% ± 3.6*
MSSA-51	100%	46.1% ± 2.4**	55.7% ± 2.4*
MSSA-84	100%	53.1% ± 3.4*	56.5% ± 4.5*
MSSA-79	100%	45.9% ± 4.1**	57.7% ± 1.4*

Hemolytic activity in untreated MSSA culture supernatant was set as 100%; values represent means ± SD (*n* = 3). *indicates *P* < 0.05 and **indicates *P* < 0.01 compared to the corresponding controls.

**Table 3a:** (a)

*S. aureus* strains	Effect of CAB extracts at BIC on MRSA surface hydrophobicity		
	Control (0)	CAB-E2 (100 *μ*g/mL)	CAB-E4 (100 *μ*g/mL)
MRSA ATCC 33591	51.60%	25% ± 3.2*	38.3% ± 2.4*
MRSA-20	62%	29% ± 3.6*	45.5% ± 3.8*
MRSA-44	27.90%	15% ± 4.1*	21.45% ± 2.8*
MRSA-45	44.20%	23% ± 3.6*	33.6% ± 3.1*
MRSA-395	29.50%	23% ± 2.6*	26.25% ± 3.4*
MRSA-410	36.50%	21% ± 2.8*	28.75% ± 3.5*

**Table 3b:** (b)

*S. aureus* strains	Effect of CAB extracts at BIC on MSSA surface hydrophobicity		
	Control (0)	CAB-E2 (100 *μ*g/mL)	CAB-E4 (100 *μ*g/mL)
*S. aureus* ATCC 11632	29.30%	15% ± 2.6*	25.3% ± 2.6*
MSSA-A8	31%	21.3% ± 3.4*	28.1% ± 3.6*
MSSA-46	33.10%	21.6% ± 2.8*	27.1% ± 4.2*
MSSA-51	20.70%	14.8% ± 3.6*	19.2% ± 3.1*
MSSA-84	22.80%	14.4% ± 2.6*	18.4% ± 2.6*
MSSA-79	31.70%	24% ± 3.1*	29.4% ± 3.4*
